# *Ligilactobacillus salivarius* regulating translocation of core bacteria to enrich mouse intrinsic microbiota of heart and liver in defense of heat stress

**DOI:** 10.3389/fimmu.2025.1540548

**Published:** 2025-04-10

**Authors:** Jiajun Yang, Peng Shang, Zongliang Liu, Jing Wang, Bo Zhang, Hao Zhang

**Affiliations:** ^1^ School of Animal Husbandry and Veterinary Medicine, Jiangsu Vocational College of Agriculture and Forestry, Jurong, Jiangsu, China; ^2^ Beijing Key Laboratory for Animal Genetic Improvement, College of Animal Science and Technology, China Agricultural University, Beijing, China; ^3^ College of Animal Science, Tibet Agriculture and Animal Husbandry College, Linzhi, China; ^4^ College of Animal Science and Technology, Aihui Agricultural University, Hefei, Anhui, China

**Keywords:** intrinsic microbiota, translocation, organs, ligilactobacillus salivarius, heat stress

## Abstract

The aim of this study was to elucidate the intrinsic microbiota residing in the heart and liver, which was enriched with Ligilactobacillus salivarius supplementation and its roles in defending anti-oxidation of heat stress. The specific pathogen free (SPF) mice were employed to perform the study. Genomic sequencing showed that the intrinsic microbes in the heart and liver of SPF mice, which were primarily of the genera *Burkholderia* and *Ralstonia*, functioned in organic metabolism, environmental information processing, cellular processes, and genetic information processing. *Lactobacillus* sp. were found in the liver but not in the heart. The heart had a lower bacterial abundance than the liver. A culturomic assay of the heart flushing liquid indicated that the dominant species of bacteria were *Ralstonia pickettii, Ralstonia* sp._3PA37C10, *Ralstonia insidiosa, Burkholderia lata*, unclassified _g_ *Ralstonia*, and unclassified _p_ *Pseudomonadota*. Intrinsic bacteria exist in the heart due to their inhibitory action against pathogenic Escherichia coli. After, the mice were supplemented with *Ligilactobacillus salivarius* to optimize the microbiota levels. The dominant bacterial phyla in the liver and heart were Bacillota, Bacteroidota, Pseudomonadota, Thermodesulfobacteriota, andActinomycetota, which comprised 98.2% of total bacteria. The genus *Lactobacillus* was also abundant. Core bacteria such as *Lactobacillus reuteri* are translocated from the intestine to the heart and liver. The enriched bacterial composition up-regulated anti-oxidation capacities in the heart and liver. The levels of reactive oxygen species and superoxide dismutase (SOD) were significantly improved compared to those in control (P < 0.01). In conclusion, intrinsic bacteria present in the heart and liver alleviate infection by pathogens, environmental and genetic information processing, and cellular processes during heat stress exposure. Diet with *Ligilactobacillus salivarius* supplementation regulated the translocation of core bacteria to the heart and liver, improved bacterial composition, and induced a higher anti-oxidative capacity under heat stress.

## Highlights

1. The intrinsic microbiota resides in the liver and heart under heat exposure conditions. The heart has less intrinsic microbiota. *Bifidobacterium longum* and *Bifidobacterium* sp. was detected in the heart without *Lactobacillus* sp. The intrinsic microbiota residing in the heart plays an inhibitory role in pathogenic *E. coli*.
2. Orally supplemented with *Ligilactobacillus salivarius* can enrich the bacterial composition in the organs of the heart, liver, and ileum and regulate the translocation of intestinal core bacteria such as *Lactobacillus reuteri* to the heart and liver, thus optimizing the richness of intrinsic bacterial composition and improving organic anti-oxidation to defend against heat stress.

## 1 Introduction

Abundant strains of bacteria inhabit the gastrointestinal tract, nasal cavity, skin, and genital tract (1). It has also been proven that microbiota exist in the lungs and trachea. The microbiota, as a second set of genomes, plays a key role in maintaining physiological homeostasis and body health (2). The connection between the intestine and other organs relies on the microbiota (3, 4), including the gut-liver axis (5), the gut-lung axis (6), and the gut-heart axis (7). A lack of evidence showing that bacteria reside in the heart and liver of healthy individuals still exists. When bodies are infected by pathogens, the heart and liver follow secondary bacterial infection (8) originated from intestinal or pulmonary opportunistic pathogens (9). Pathogenic bacteria can be detected in the hearts and livers of dead animals, especially during farming animal production (10). Chickens and weaned piglets are infected or secondary infection with pathogenic *E*. *coli*, which can be detected in the heart and liver (11). Whether the intrinsic microbiota existing in the liver and heart constitutes the microbiological barrier to defend against pathogenic infections need to be disclosed in healthy conditions. Once the existence of bacteria in the organs of the liver and heart is proven, their function is waiting to be unveiled, especially in defense against pathogenic infection.

*Ligilactobacillus* spp. and certain strains of lactic acid bacteria are often used in biomedicine and animals breeding (12). *Ligilactobacillus salivarius* and *Lactobacillus* reuteri are the core bacteria residing in the gastrointestinal tract, play crucial roles in immunity and anti-oxidation, and are housekeeping bacteria in the host (13, 14).

In summer, the temperature can reach more than 37 °C especial in south of China, which cause heat stress to both people and animals. Under heat stress conditions, appetite is reduced, and the disorder in hormone secretion induces a decline in immune levels, leading to sub-health conditions for the body. In the gut, strains of pathogens and opportunistic pathogens tend to cause endogenous infections under sub-health conditions. Do the core bacteria trans-locate its residing place to other organs during bodily stress?

To address these two questions, we used laboratory mice, an experimental model widely used in biological studies (15). Two animal assays were performed in this study. The first one was to verify the existence of intrinsic microbiota in the heart and liver. Mice were raised to verify the presence of intrinsic microbiota in the heart, liver, and lungs. Flushing liquid from the liver, lungs, and heart was prepared to test the possibility of bacterial existence using metagenomic sequencing. The presence of bacteria in the heart was detected using culturomic techniques to discern the differences in bacterial composition, and the inhibitory action on the pathogen was evaluated. Secondly, mice raised under heat exposure were orally supplemented with *Ligilactobacillus salivarius* to optimize the composition of the microbiota in both the heart and liver. The translocation of core bacteria from the intestine to the heart and liver to defend against heat stress was measured. This study aimed to unveil the intrinsic microbiota residing in the heart and liver under healthy conditions. Supplementation with *Ligilactobacillus salivarius* can enroll the translocation of core bacteria to optimize cardiac and hepatic bacterial composition and defend against oxidation in mice under heat stress. Our study reveals a new connection between the intestine, heart, and liver.

## 2 Materials and methods

### 2.1 Mouse, antibiotics and samples harvesting

Seventy-two specific pathogen free male BALB/c mice weighing 16g were purchased from the Animal Center of the Nanjing General Hospital of Chinese People’s Liberation Army. The mice were housed in JV222 IVC cages under a 12–12-h dark–light cycle with food pellets and water ad libitum. The room temperature maintained 20-22 °C. All animal experiments complied with the ARRIVE guidelines and were conducted in accordance with the U.K. Animals (Scientific Procedures) Act, 1986 and the National Research Council’s Guide for the Care and Use of Laboratory Animals. All experimental protocols used in this study, including animal husbandry and slaughter, were approved by the Institution of Animal Science and Welfare of Jiangsu Province (No. JSIASWAP2022070393).

The treatment of the mice was showed in **Supplementary Table 1**. After adaptive feeding for 7 d, 10 average body weight (24.76 ± 0.43g) in total of 72 mice was selected and sacrificed by cervical dislocation in a bio-safety cabinet. The organs of heart, lung, and liver were sampled. Organs were cut into small pieces and allocated in 2mL sterilized tubes to prepare the flushing liquid. One copy of the flushing liquid from the heart was disposed of to perform culturomic analysis.

### 2.2 Sample processing and metagenomic sequencing

A Samples of the lung, heart and liver (0.2 g) were immersed in 20mL sterilized ice cold double distilled water in a 9 cm bacteria-free plate for 20 min, and the tissues were flashed with the immersed liquid. Then flushing soak was harvested and precipitated in 5000 rpm/min for 10 min to collect the bacterial cells. To increase the harvesting production of cells, the supernatant was re-used to flush the tissues, and employed with a repeated precipitation (5000 rpm/min for 10 min). The pellet was resuspended in Tris-HCl (50mM, pH 6.8) to extract total DNA. A volume of 1mL of the cardiac flushing liquid was centrifuged at the speed of 5000rpm/min to get the sediment. Sediments were dissolved in Tris-HCl for DNA extraction.

One total of 1 μg DNA per sample was used as the input material for the DNA sample preparation. Sequencing libraries were generated and enriched using a reaction of polymerase chain reaction (PCR). TruSeq™ DNA Sample Prep Kit (Abcam, USA) was used to extract the total samples DNA following the manufacturer’s protocol, which were fragmented by sonication to a size of 300 base pairs. The DNA fragments were end-polished, A-tailed, and ligated with a full-length adaptor as a bridge for PCR amplification (HiSeq 3000/4000 PE Cluster Kit). Finally, the PCR products were sequenced using polymerase and four fluorescently labeled base pairs (HiSeq 3000/4000 SBS Kits). After cluster generation, the library preparations were sequenced on an Illumina HiSeq platform (16). The low-quality raw reads containing N reads in the primary sequencing data were filtered to obtain a clean read. MEGAHIT (v1.1.2 https://github.com/voutcn/megahit) and CD-HIT software (v4.6.1 http://weizhongli-lab.org/cd-hit/) were used. To remove interference originating from host genetic reads, BWA software (v0.7.17 http://bio-bwa.sourceforge.net/) was used to compare with the Ensembl Release 104 database (https://ensembl.org/index.html). The Software SOA Paligner (soap2.21release https://github.com/ShujiaHuang/SOAPaligner) was used to align the nucleotide sequences. Beta diversity distance measurements were performed with weighted UniFrac to investigate the structural variation in microbial communities across samples, and then visualized via principal coordinate analysis (PCoA) and principal component analysis (PCA). In addition, Clusters of Orthologous Groups (COG, 2020; http://eggnog5.embl.de/#/app/downloads) was used to predict the function of the microbiota in the organs.

The raw Illumina sequencing data of the heart, liver, and lungs were deposited in the Sequence Read Archive database (SRP) of NCBI (SRR29213176, 29234301, 29240348). The BioProject accession numbers were PRJNA1117782, 1118213, and 1118345.

### 2.3 Culturomics assay on flushing liquid of heart

The classic culture method combined with sequencing was used to verify the presence of lactic acid bacteria in the heart (17). Sterilized de Man, Rogosa, and Sharpe (MRS) broth and Yeast Extract Peptone Dextrose (YEPD) medium were prepared (18). A volume of 0.1 mL of flushing liquid of heart were cultured in MRS broth and YEPD medium. After cultured for 18 h, 0.1 mL of the fermented liquids was soaked to test its inhibitory action on pathogenic *E*. *coli* 25922 (purchased from Central Microorganism Storage, Beijing, China). *E*. *coli* 25922 cells were cultured in Luria-Bertani (LB) broth. Then, 0.1mL of the bacterial fluid was spread evenly onto plates containing MacConkey solid culture medium. The inhibitory action of the fermented liquids was measured using the cylinder-plate method (19). Then, the plates were laid in facultative anaerobic cultivation for 20 h at 37 °C to observe the flora of bacteria. The composition of bacteria in the heart and their fermentation in the MRS were measured using metagenomic sequencing. The pH of fermentation in the MRS was also tested.

Raw Illumina sequencing data of the heart were deposited in the Sequence Read Archive database (SRP) of NCBI (SRR29254823). BioProject accession number: PRJNA1119040.

Through the assays described in the first section, the intrinsic bacteria existing in the heart and liver and their potential function in the inhibition of pathogenic E. coli 25922 (purchased from Central Microorganism Storage, Beijing, China) were detected. To further evaluate the role of *Ligilactobacillus salivarius* in modulating the translocation of gastrointestinal core bacteria to the liver and lungs under heat stress, another animal experiment was conducted.

### 2.4 Probiotic cultivation, and mice treatment

*Ligilactobacillus salivarius* was isolated by our research group at Jiangsu Vocational College of Agriculture and Forestry. It was stored at the China General Microbiological Culture Collection Center (CGMCC), Beijing, China (storage number CGMCC17718). The bacteria was inoculated in MRS medium following 1% inoculum size, cultured in 37 °C for 16 h. The live number of bacteria reached 1.6×10^10^ colony forming units per milliliter (CFU/mL).

Forty mice with an average body weight of 20 g were used in this study. Twenty mice were allocated to *Ligilactobacillus salivarius* supplemented group, and another 20 mice were fed as the control indicated in **Supplementary Table 2**. There were four replicates of each group, with five mice per group. Feed and management were in agreement with those described in a previous trial. Mice were housed in an air conditioned room at 37 °C to create heat stress. In the supplemented group, mice were infused with 0.4 mL fermentation liquid of *Ligilactobacillus salivarius*. In the gavage administration of 20 g weight of mice, the volume of infusion cannot exceed 0.5mL. Hence, a volume of 0.4mL is suitable. After *Ligilactobacillus salivarius* cultured, the fermentation solution was diluted with saline until the live number reached 1×10^8^ CFU/mL according to the suggested dosage (20).

After feeding for 7 d, two mice with average body weights of 27.93 ± 0.39 g were selected from each replicate. The heart, liver, and segment of the ileum of eight mice from each group were harvested in an aseptic environment.

### 2.5 Sample preparation, DNA extraction and polymerase chain reaction

Samples (0.2 g) of the liver and heart were cut into small pieces and washed with a flushing liquid, as previously described. The flushing liquid was then harvested. The ileal samples were cut into 2-cm pieces, which were spread out, and the mucosa was scraped from the intestine with sterilized slides after the contents had been washed with Tris-buffered saline containing 0.1% Tween 20 to extract DNA. The total DNA was extracted using the E.Z.N.A.^®^ tissue DNA Kit (Omega Bio-tek, Norcross, GA, USA) according to the manufacturer’s protocol. DNA concentration and purity were determined using a NanoDrop 2000 UV-vis spectrophotometer (Thermo Scientific, Waltham, MA, USA), and DNA quality was determined by 1% agarose gel electrophoresis. The V3-V4 hypervariable regions of the bacterial 16S rRNA gene were amplified with primers 338F (5-ACTCCTACGGGAGGCAGCAG-3′ and 806R (5′-GGACTACHVGGGTWTCTAAT-3′) using a thermocycler PCR system (GeneAmp 9700, Applied biosystems, Foster City, CA, USA). Polymerase Chain Reaction (PCR) was conducted as follows: 3 min of denaturation at 95 °C, 27 cycles: 30 s at 95 °C, 30 s of annealing at 55 °C, 45 s of elongation at 72 °C, and a final extension at 72 °C for 10 min. PCR was performed in triplicate in 20-μL mixtures containing 4 μL of 5×FastPfu Buffer, 2 μL of 2.5 mM dNTPs, 0.8 μL of each primer (5 μM), 0.4 μL of FastPfu polymerase, and 10 ng of template DNA. The resulting PCR products were analyzed by electrophoresis on a 2% agarose gel to detect the copies of bacteria in different samples (21).

### 2.6 16S rDNA sequence and analysis.

The PCR products were extracted from a 2% agarose gel and further purified using the AxyPrep DNA Gel Extraction Kit (Axygen Biosciences, Union City, CA, USA) and quantified using QuantiFluor™-ST (Promega, Madison, WI, USA) according to the manufacturer’s protocol. Purified amplicons were pooled at equimolar concentrations and paired-end sequencing was performed (2 × 300) on an Illumina MiSeq platform (Illumina, San Diego, CA, USA) according to standard protocols. Raw Illumina sequencing data were deposited in the Sequence Read Archive database (SRP) of NCBI SRR18190482. The BioProject accession number is PRJNA811797. Diversity metrics were calculated using the core diversity plugin in QIIME2 (21). The alpha diversity was conducted through pan/core analysis. The index of operational taxonomic units (OTUs) were used to estimate the microbial diversity within an individual sample. In addition, potential Kyoto Encyclopedia of Genes and Genomes (KEGG) (22) ortholog functional profiles of the microbial communities were predicted using PICRUSt. Raw Illumina sequencing data were deposited in the Sequence Read Archive database (SRP) of NCBI (SRR18190482). The BioProject accession number is PRJNA811797.

### 2.7 Assay for fluorescence *in situ* hybridization and quantitative PCR

For mice supplemented with *Ligilactobacillus salivarius* supplementation, the heart, liver, and ileum were harvested. Translocation of *Lactobacillus reuteri* to the heart and liver was investigated using FISH (23). The probe was designed based on the 16S ribosomal sequence of *Lactobacillus reuteri* (24), which was isolated and stored at the China Center for Type Culture Collection (CCTCC), Wuhan University, Wuhan, China (strain number CCTCC M2015660). Probes with carboxytetramethylrhodamine were designed and conjugated to the DNA of *Lactobacillus retuteri* with sufficient length to ensure specific binding (**Supplementary Table S1**). Organs (0.2 g) such as the liver, heart, and ileal mucous membrane were harvested and homogenized with 1.8mL ice cold saline, and 50μL of homogenate was fixed by immersion in 2mL 10% formaldehyde for 24 h, which was transferred to poly-L-lysine-coated slides and air-dried on a sterile benchtop for 3 h. Samples were then incubated with lysozyme at 32 °C for 10 min, washed with distilled water, immersed in 70% ethanol for 2 min, and air-dried. The probe was diluted to 60 nM before use. 12 μL of probe were then added to the tissue, followed by incubation at 46 °C for 12 h, and washed with phosphate buffer solution (pH 7.4). The tissue was stained with 4′,6-diamidino-2-phenylindole for 5 min and washed thrice with distilled water for 5 min each. After drying, the slides were mounted using Fluoromount-GTM (Abcam, Cambridge, UK) and observed under a fluorescence microscope (BX53; Olympus, Tokyo, Japan).

Strain of *Lactobacillus reuteri* was cultured in beef extract peptone medium and a tenfold dilution series of *Lactobacillus reuteri* was performed. The CFU of *Lactobacillus reuteri* were counted using the plate method under a microscope to obtain samples of 1 × 10^4^, 10^5^, 10^6^, and 10^7^. Total RNA in each dilution was extracted using the RNA Extraction Kit (Invitrogen, Carlsbad, CA, USA). Reverse transcription was performed using the GoScript Reverse System (Invitrogen). Primers were designed according to the 16S ribosomal RNA of *Lactobacillus reuteri*, and are listed in **Supplementary Table 3**. Quantitative PCRs was performed using SuperReal PreMix Plus (SYBR Green, FP205) on an ABI 7900HT Fast Real-Time PCR System. Relative expression levels of target genes were quantitatively normalized against the expression of β-actin using the 2^-ΔΔCT^ method. First-strand cDNA was synthesized by incubating a reaction mixture containing 11 μL RNA and 1 μL RNase-free dH2O at 70 °C for 3 min, followed by 0 °C for 5 min. The cDNAs was also used for PCR. A dNTP mixture (1 μL; 10 mmol/l), 4 μL GoScript 5X reaction buffer, 1 μL GoScript reverse transcriptase, 1.5 μL Mg^2+^ (25 mM), and 0.5 μL RNase inhibitor were combined in a total volume of 20 μL and incubated at 37 °C in a water bath. A standard curve was established based on the number of *Lactobacillus reuteri* and CT values. Samples of the ileum, heart, and liver from mice with and without *Ligilactobacillus salivarius* supplementation were harvested. 0.15 g sample was used to extract total RNA, and qPCR was performed as described above. The levels of *Lactobacillus reuteri* in the samples were converted to real numbers using a standard curve.

### 2.8 Assay for anti-oxidation indexes

Samples of the heart, liver, and ileal mucosa (0.15 g) were weighed and placed in 5 mL sterilized tubes. Tissues were incubated and digested with 100 U/mL collagenase (Sigma, USA) buffer (20 μg/mL neutral protease II, 32 mM HEPES, 127.5 mM NaCl, 3.15 mM KCl, pH 7.60, 37 °C) for 60 min. The cell suspension was filtered through mesh (grid size about 150 μm) and further ten diluted with1.35 mL ice cold saline to collect the cellular samples for reactive oxygen species (ROS) and superoxide dismutase (SOD) assays.

For ROS test (25), a volume of 30 μL cellular sample was sucked and added into 270 μL dimethyl sulfoxide dilution (10mM) contained 5 μmol/L 2,7-dichlorofuorescin diacetate as a fluorescence primer can be hydrolyzed into dichlorofluorescin in cells and were oxidized in strong green fluorescence. Mix the samples and reagent, and incubate the tubes in 37 °C for 30 min. The tubes were centrifuged at 5, 000 rpm/min for 5 min, and the supernatant was removed. The precipitate was resuspended in ice-cold, double-distilled water. 200 μL liquid was added into 96 well plate for fluorescence detection for excitation and emission wavelength was 488 nm and 525 nm respectively. The results are shown as fluorescence values.

For SOD measurement (20), a volume of 30 μL cellular sample was sucked and added into 270 μL 0.01 mol/L phosphate buffer solution. The manufacturing protocol followed the instructions of the testing kit (Nanjing Jiancheng Bioengineering Institute, China).

### 2.9 Statistical analyses

Body weight, qRT-PCR, and sequencing data were subjected to one-way ANOVA using the GLM procedure in SPSS, with significance reported at P < 0.05. The correlation of bacterial composition with anti-oxidation was measured with Spearman’s analysis (R,version 3.3.1, pheatmap package).

## 3 Result

### 3.1 Intrinsic bacteria existence in organs and its functions

**Figure 1:**
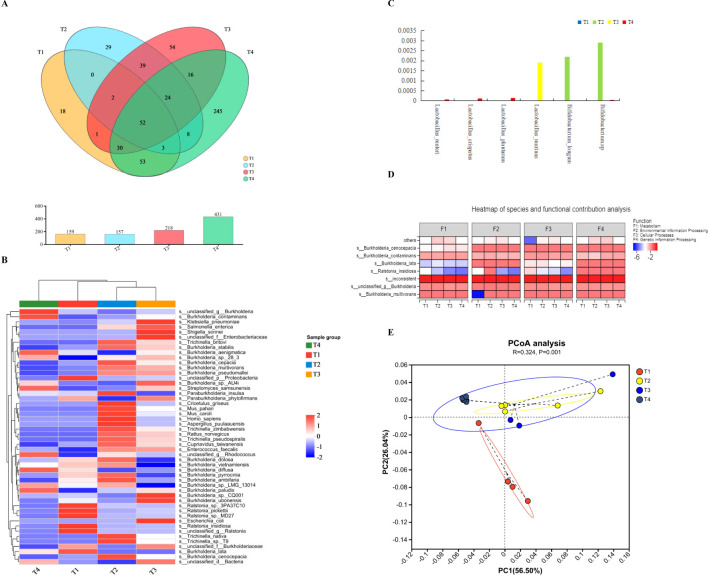
Bacterial composition in the hearts and flushing liquid cultivation, livers, and lungs of mice. **(A)** The Venn diagram shows the bacterial OTU in T1–T4 representing pure cultivations of cardiac flushing liquid, hearts, livers, and lungs, respectively. The number in the colored circles and overlapped circles represent the owned OTU in one or more groups and the correlated proportion, respectively. The bacterial OTU in the lungs was most abundant during heat stress and that in the heart was the lowest. **(B)** The differences in bacterial species levels with different treatments (T1–T4: pure cultivation of cardiac flushing liquid, hearts, livers, and lungs). **(C)** Presence of *Lactobacillus* sp. and *Bididobacterium* sp. with different treatments. **(D)** Analysis on bacterial species and functional contributions. “F” indicates function, and F1–F4 represent metabolism, environmental information processing, cellular process, and genetic information processing respectively. **(E)** PCOA analysis on the bacterial composition and functions of organs. UniFrac-based PCoA on similarities with function revealed significant differences in the bacterial community structure among groups (illustrated in the PCoA plot). Two primary factors reflect the function of heart, its cultivation, liver, and lungs. The differences of organs and use of antibiotics would be two factors which components 56.50% and 26.04% in primary proportion, respectively.

The intrinsic microbiota can be detected in the heart and liver of mice. After removing the genetic interface from the host, 842, 179, and 176 valid clean reads were harvested comprising 99.39% of the total genome. The heart had the lowest richness of bacteria; only 157 OTUs were detected, and 159 OTUs were found in the cultivation of cardiac flushing liquid, which was both lower than those in the liver and lungs, with 218 and 431 OTUs respectively manifested in **Figure 1A**. The overlapping parts of the OTUs between the lungs and liver were greater than those between the lungs and cardiac flushing liquid. At the genus level, *Burkholderia* was the primary bacterial species found in the heart, liver, and lungs. However, the bacterial composition of the liver differed. Some strains of *Klebsiella pneumoniae*, *Shigella sonnei*, and unclassified *Enterobacteriaceae* were also detected in the liver. In addition to more strains of *Streptomyces samsunensis*, unclassified _g_ *Rhodococcus* was found in the lungs treated with streptomycin and ampicillin (**Figure 1B**). The sequencing data further validated strains of *Lactobacillus* sp., namely *Lactobacillus reuteri*, *Lactobacillus crispatus*, and *Lactobacillus plantarum* only residing in the lungs. And *Lactobacillus murinus*, which exists only in the liver. *B*._*longum* and *Bifidobacterium* sp. only resided in the heart showed in **Figure 1C**. The species of *Burkholderia* and *Ralstonia* in organs function in organic metabolism, environmental information processing, cellular processes, and genetic information processing are shown in **Figure 1D**.

UniFrac-based principal coordinate analysis (PC_O_A) on similarities with function revealed significant differences in the bacterial community structure among the groups (**Figure 1E**). The bacterial composition and use of antibiotics were the two primary factors, accounting for 56.50% and 26.04%, respectively, reflecting the functions of the heart, liver, and lungs.

### 3.2 The cultivation of microbiota in heart and its inhibitory action on pathogen

**Figure 2:**
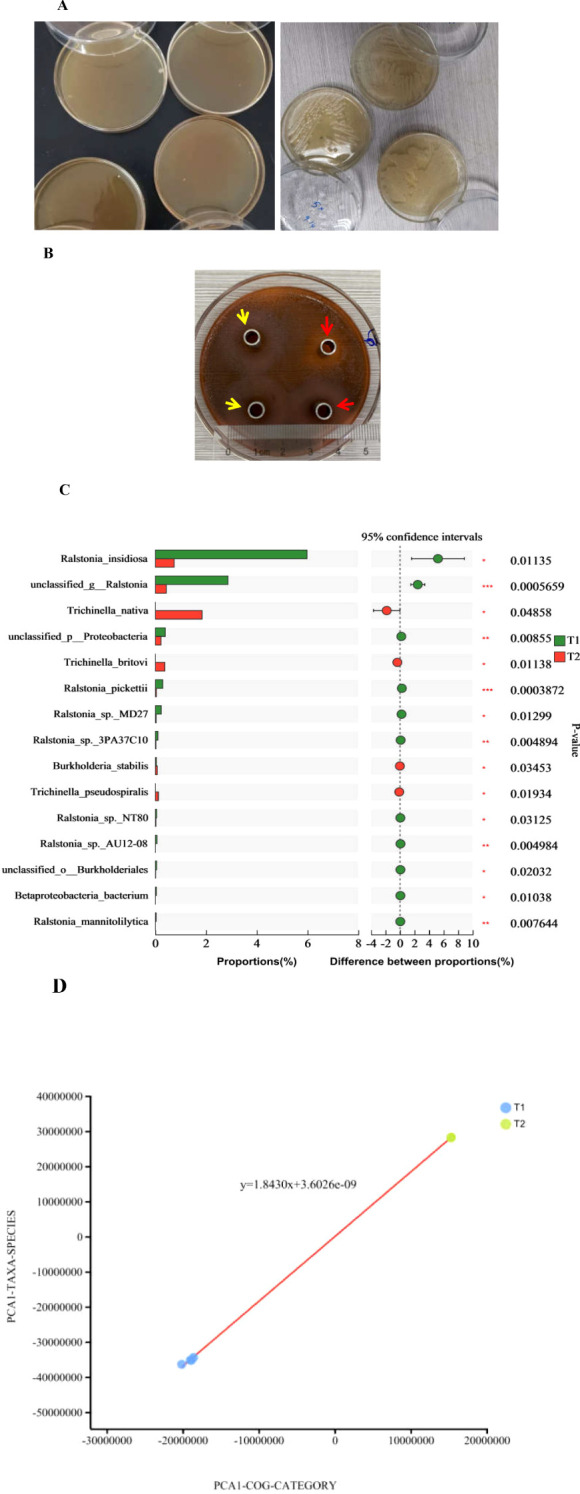
Bacterial composition and functional comparison between the heart and its cultured samples. **(A)** Bacterial colonies on plates measured using culturomics. Heart samples of mice with antibiotic supplementation was removed and flushed, then cultured in MRS and YEPD broth for 16 h. Next, the cultural liquid was inoculated in MRS and YEPD plate medium and cultured for 36 h in facultative anaerobic conditions. Bacterial colonies were detected on the plate. **(B)** Inhibitory action of cardiac flushing liquid cultivation in MRS and YEPD medium on pathogenic *E*. *coli* 25922. The cardiac flushing liquid culture was added to a cylinder cup to detect the inhibitory action on pathogenic *E*. *coli* 25922. The two left zones were produced by liquid cultivation in MRS indicated by yellow arrows. The diameter of inhibitory zones reached 20.39 mm. The two right zones were produced by liquid cultivation in YEPD medium indicated by red arrows. The diameter of inhibitory zones reached 18.89 mm. **(C)** Comparison of the bacterial composition of the heart and its cultured samples. Cardiac flushing liquid was cultured in MRS medium. Presence of bacteria in the heart and its cultivation was measured using metagenomics sequencing. *, *P* < 0.05; **, *P* < 0.01; ***, *P* < 0.001. **(D)** The PCA on correlation of bacterial composition with function between samples of the heart and its cultivation. Functions were predicted and contrasted with COG of proteins collected in the NCBI. Every COG family comprised at least three phylogenetic lineage proteins. COG function and species of bacteria are in accordance with the abscissa and ordinate. The regression equation was established. 3.6026e-09 indicates 3.6026×10^-9^.

Culturomics techniques were used to unveil the dominate species of bacteria contained in heart (**Figure 2A**) for scarce bacteria. The most cultivable microorganism were genus of *Burkholderia* manifested on an MRS plate. The pH value of the culture in the MRS medium reached 3.5. Intrinsic bacteria reside in the heart because of their inhibitory action on pathogenic *E*. *coli*, as shown in **Figure 2B**. *Burkholderia* and *Ralstonia* were the two primary genera in the cultivation of cardiac flushing liquid. Strains of *Ralstonia*_*pickettii*, *Ralstonia*_*sp*._*3PA37C10*, *R*._*insidiosa*, *Burkholderia*_*lata*, unclassified _g_ *Ralstonia*, and unclassified _p_ *Pseudomonadota* in heart was accounted for most of the population in the heart. After cultivation, additional strains of *Burkholderia stabilis*, *Trichinella britov*i, and *Trichinellanativa* were detected (**Figures 1B**, **2C**). Cultivated nutrition strictly required bacteria; *B*. *longum* and *Bifidobacterium* sp. were unidentified (**Figure 1C**). The bacterial function in the culturomics of heart-flushing liquid indicated fewer roles in environmental information processing, cellular processes, and metabolism compared with the flushing liquid of the heart (**Figure 1D**). This function strongly correlated with the bacterial composition of the heart and its cultivation, as shown in the PCA plot (**Figure 2D**).

### 3.3 The profile of bacterial composition of mice with *L*. *salivarius* supplementation

**Figure 3:**
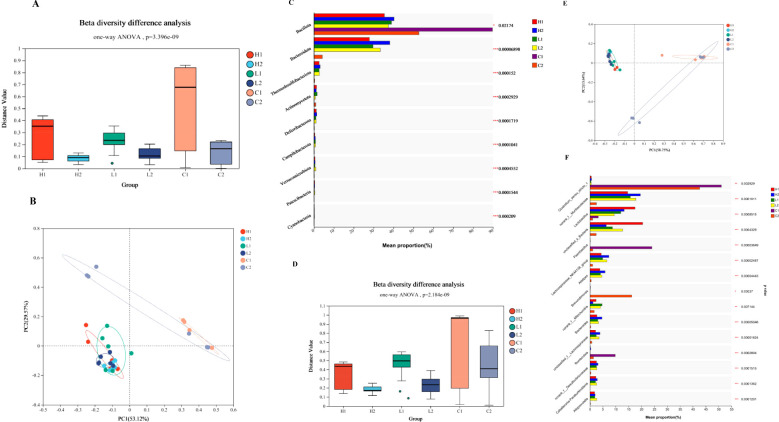
Profiles of bacterial composition in organs of control and *Ligilactobacillus salivarius* supplemented mice (H1, L1, and C1 represents the heart, liver, and ileal mucosa of *Ligilactobacillus salivarius* supplemented mice, respectively; H2, L2, and C2 represents the heart, liver, and ileal mucosa of control mice, respectively). **(A)** Bacterial richness of β diversity at the phylum level. P=3.396e-09 indicates 3.396×10^-9^. **(B)** PCoA of the bacterial differences at the phylum level. The figure was constructed using ANOSIM of the UniFrac distance metric. The distances between different colored points represent the relation of bacterial composition in treatments. **(C)** Bacterial composition of various treatments at the phylum levels. **(D)** Bacterial richness of β diversity at the phylum level. P=2.184e-09 indicates 2.184×10^-9^. **(E)** PCoA of the bacterial differences at the genus level. **(F)** Bacterial composition of different treatments at the genus levels.

Mice with *Ligilactobacillus salivarius* supplemented for 7 d under heat conditions, then the intrinsic bacterial composition of the heart, liver, and ileal mucosa were measured using 16S rDNA sequencing. A total of 1, 003, 673 raw reads were obtained in total. After removing the low-quality sequences, 368, 213 clean tags were identified. Mice with *Ligilactobacillus salivarius* supplementation the bacterial composition of the heart, liver, and ileum improved at the phylum level indicated in **Figure 3A**. Principal coordinates analysis (PCoA) indicated that two primary factors accounted for 82.69% of all attributes of the differences in bacterial composition at the phylum level (**Figure 3B**). Five phyla: Bacillota, Bacteroidota, Pseudomonadota, Thermodesulfobacteriota, and Actinomycetota, accounted for nearly 98.2% of the total bacterial composition (**Figure 3C**). The richness of the phyla Bacillota and Bacteroidota was reduced, and more phylum of Pseudomonadota was replaced with a reduced proportion. The bacterial composition at the genus level was also rich upon supplementation with *Ligilactobacillus salivarius* (**Figure 3D**). The dispersed area indicated that the bacterial composition was more uniform. The main factors contributed to 72.39% of the differences in composition (**Figure 3E**). The richness of the genera *Lactobacillus* (P<0.001), *Paenibacillus* (P<0.001), and norank_f_*Mitochondria* (P<0.01) in heart, liver, and ileal mucosa were all increased (**Figure 3F**). The richness of unclassified_k:norank_d:Bacteria in the heart also improved, whereas it decreased in the liver and ileum (P<0.001). The proportion of the genera *Alistipes*, norank_f_ *Muribaculaceae*, and *Bacteroides* were reduced in all three organs of *Ligilactobacillus salivarius* supplemented mice (P<0.001).

### 3.4 Correlation of anti-oxidation with bacterial strain difference

**Figure 4:**
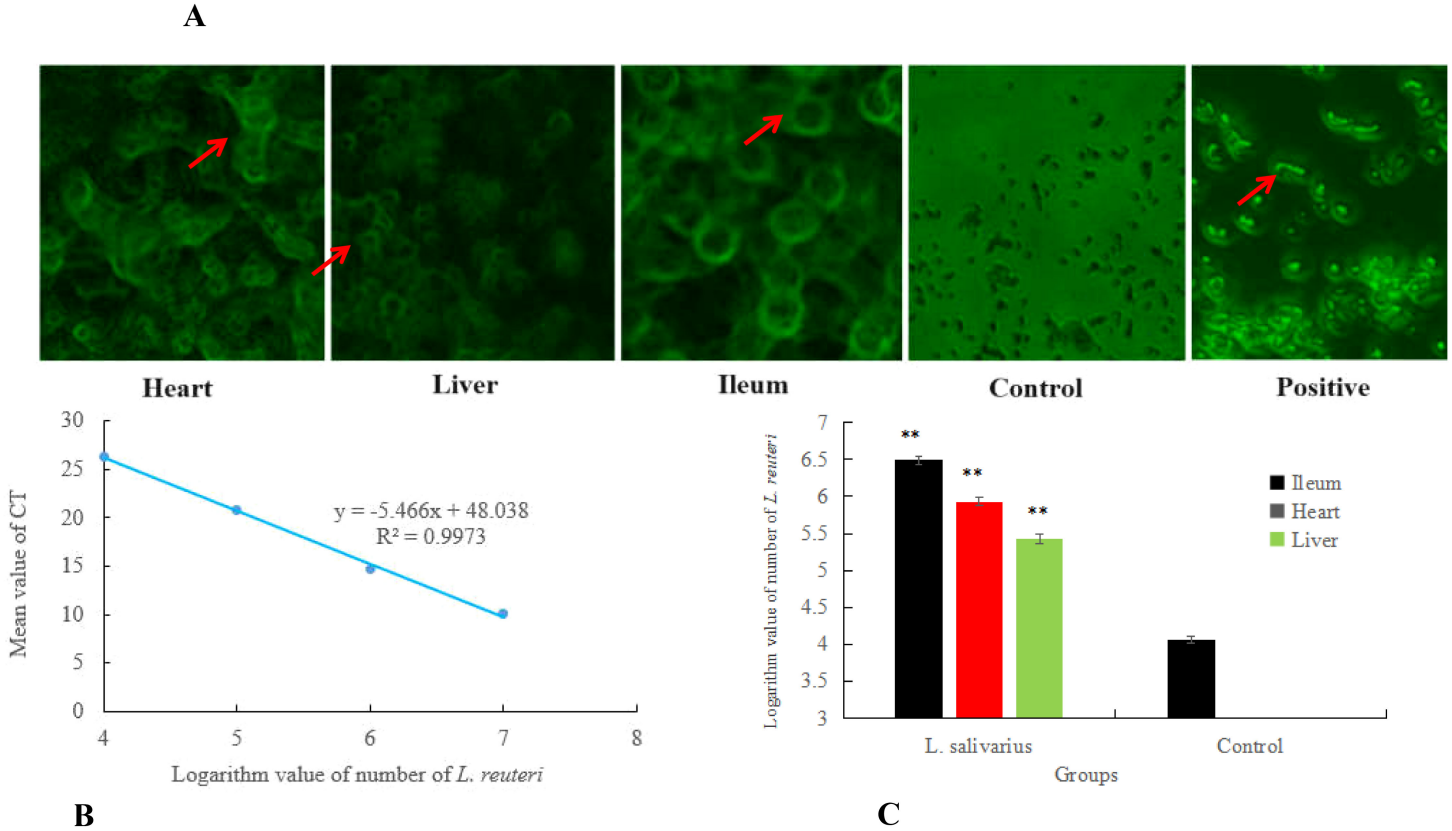
Translocation of *Lactobacillus reuteri* in hearts and livers of mouse exposure to heat. **(A)** Real-time qPCR assay on the number of *Lactobacillus reuteri*. Colonization of *Lactobacillus reuteri* in the heart, liver and ileum was detected with FISH. **(B)** Total number of *Lactobacillus reuteri* estimated based on a standard curve by qPCR assay. Standard curve of qPCR assay on series of diluted *Lactobacillus reuteri* (10^4^, 10^5^, 10^6^, 10^7^ dillution). **(C)** Total number of *Lactobacillus reuteri* estimated based on standard curve. Values represented by vertical are means, with standard errors are represented by vertical bars. ** means P<0.01.

The anti-oxidative capacity in heart, liver and ileum was measured shown in **Figure 4A**, both in tissue of heart and liver, the level of ROS produced (in per second) in *Ligilactobacillus salivarius* supplemented mice was lower than control mice (P < 0.01). The levels of produced ROS were also reduced in the ileum following *Ligilactobacillus salivarius* supplementation (P < 0.01). SOD activity in the heart, liver, and ileum was significantly higher than that in control (P < 0.01). The differences in microbial composition at the species level showed that more strains of *Lactobacillus reuteri* (P < 0.001), *B*. *stabilis* (P < 0.001), and *Ralstoniapickettii* (P < 0.01) became richer than in the control in all three organs (**Figure 4B**). *Lactobacillus reuteri*, *Lactobacillus murinus*, *Lactobacillus johnsonii* owning strong correlation with anti-oxidative parameters. Some bacterial species, *Lachnospiraceae*_*bacterium*, uncultured_*bacterium*_g:norank_f:*Muribaculaceae*, uncultured_*Muribaculaceae*_*bacterium*, unclassified_g:*Muribaculum*, and norank_f_ *Mitochondria* also play significant roles in the defense against heat stress (**Figure 4C**).

### 3.5 Translocation of core bacteria in heart and liver

**Figure 5:**
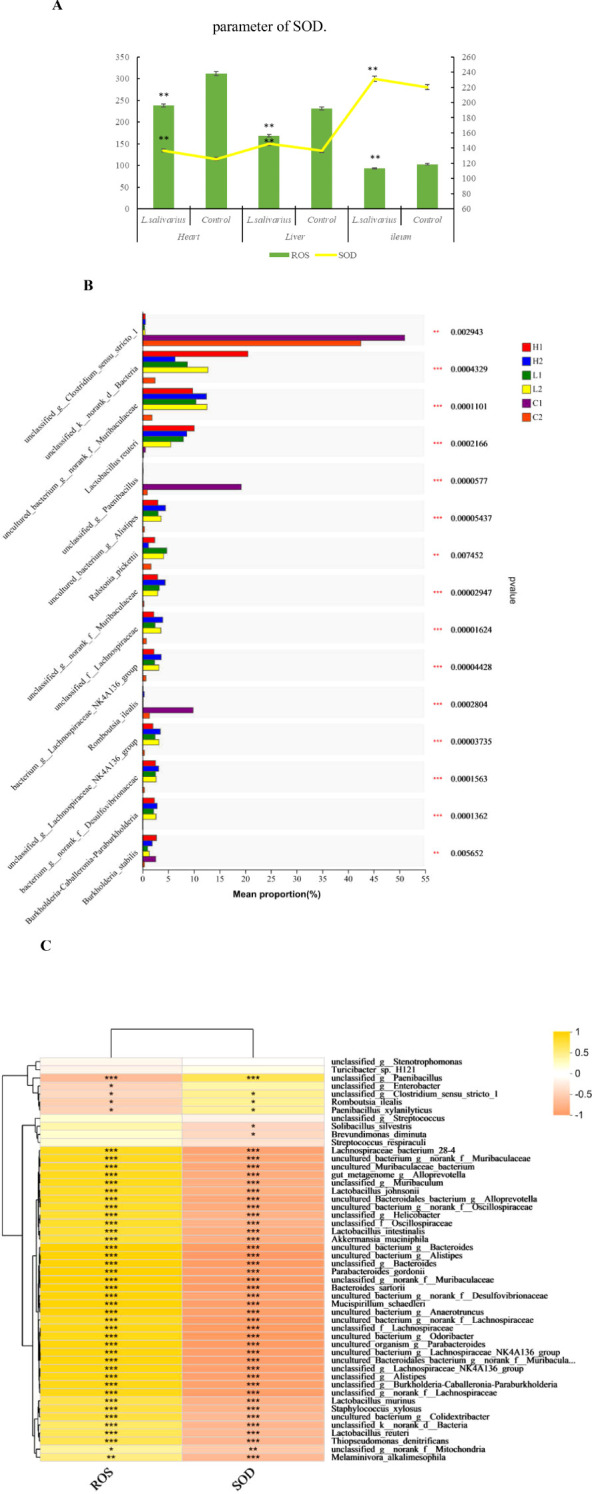
Correlation of anti-oxidation with bacterial differences at the strain level. **(A)** Anti-oxidation level in different treatments. The left abscissa is the SOD parameter. * in the same area indicates P < 0.05. ** in the same area indicates P < 0.01. **(B)** Bacterial differences at the strain level. Typical bacterial strains in the livers, hearts, and ileal mucosa of mice with *Ligilactobacillus salivarius* supplementation. Richness of the *Lactobacillus reuteri*, strain of *B*. *stabilis* and *Ralstonia_pickettii*. **(C)** Heatmap of Spearman’s correlations between the significantly modified strains of bacteria with antioxidative parameters in mice. Asterisks in different colors represent significant positive correlations. ***indicates very strong correlation. Distinct strains of *Lactobacillus reuteri*, *Lactobacillus murinus*, and *Lactobacillus johnsonii* showing strong correlation with anti-oxidative parameters.

Under heat stress, oral supplementation with *Ligilactobacillus salivarius* significantly increased the richness of *Lactobacillus* in the heart, liver, and ileum (**Figure 3F**) and increased the proportion of *Lactobacillus reuteri* (**Figure 4B**). To further validate the translocalization of core bacteria in the heart and liver, FISH and qPCR assays were performed, and the results are shown in **Figure 5A**. More *Lactobacillus reuteri* 16S fluoresce-labeled green spots were found in the homogenates of the heart, liver, and ileum. qPCR results indicated that the number of *Lactobacillus reuteri* Reached 10^5^ CFU/g in both the heart and liver of mice supplemented with *Ligilactobacillus salivarius* supplementation, showed in **Figures 5B, C**. However, a low number of *Lactobacillus reuteri* was found in the control mice.

## 4 Discussion

In animal production, the indigenous pathogenic infection often occurred in heart and liver, the pathogen *Escherichia coli* or *Salmonella* can be detected. In condition of low bodily immunity, the opportunistic pathogenic bacteria often trans-locate from large intestine to small intestine or other organs of liver, heart, or lung, which was secondary infection (26). Heat stress influences body health in both humans and livestock by reducing immune levels and anti-oxidative capacities (27). Microbiota in the GIT is influenced by bodily stress conditions (28). Indigenous opportunistic pathogens propagate easily. These surplus microbiota are potential threats that translocate to other organs to induce further damage during the development of stress and pathogenic infections (29).

Whether intrinsic microbiota is present in the heart and liver remains unknown. In addition, studies are required to determine whether the intrinsic microbiota residing in the heart and liver play a role in defending against pathogenic *E*. *coli* infections. A previous study suggested that none of the microbiota exists in the heart and liver under health condition (30, 31). It is important to advance our understanding of bacterial presence in the body. Mice raised in heat stress condition were to identify these intrinsic bacteria in heart and liver. Results of whole-genome sequencing on the flushing liquid of the tissues indicated that intrinsic bacteria can be found both in the liver and heart.

In terms of bacterial composition, the shared parts of the heart and liver were more abundant than those of the lung. The gut-liver axis bridged by the microbiota can reach the liver through the portal vein. Macrophages in the sinusoids of the liver detoxify toxins and pathogens in the gut. Thus, only a few pathogens were detected in the liver. The genera *Burkholderia* and *Ralstonia* were the predominant bacteria in the heart, liver, and lungs. Typical bacteria are found in specific organs. Among tissues of heart, liver and lung, strains of *Lactobacillus reuteri*, *Lactobacillus crispatus*, and *Lactobacillus plantarum* reside only in the lungs, whereas *Lactobacillus murinus* exists only in the liver. *B*._*longum* and *Bifidobacterium* sp. are typically detected in the heart. The genera *Lactobacillus* and *Bifidobacterium* are the two primary core bacteria in the intestine (32), and some species have housekeeping functions inherited from ancestors (33, 34). Some strains of these core bacteria reside in the heart, liver, and lungs as intrinsic microbiota and function in information processing, organic metabolism, and immunity (35, 36). In the lungs, *S*. *samsunensis* is a potential antibiotic that produces bacteria to defend against pathogenic bacteria infection (37). For the lowest abundance of bacteria in the heart, the flushing liquid was cultured and sequenced to determine its ability to ravel intrinsic bacteria. Cultivatable microorganisms that primarily belong to the genus *Burkholderia* which strongly inhibits pathogenic *E*. *coli*. The genera *Burkholderia* and *Ralstonia* account for the majority of the microbiota of the heart after cultivation compared with those without, especially the genus of *Ralstonia*, which account for nearly 75% of the total. This bacterium functions in the defense of the pathogen (38). The most abundant cultivatable microorganisms were strains of *B*. *stabilis*, *T*. *britovi*, and *T*. *nativa* owing to their inhibitory role on pathogenic *E*. *coli*. Owing to the shortage of cultures, *B*. *longum* or *Bifidobacterium* sp. could not be detected because of their strict nutritional requirements (39), which prevented them from propagating in MRS medium. Whole genome sequencing data showed that *Lactobacillus reuteri* is an intrinsic bacterium in the lungs that cannot be detected in the heart or liver. The nutrition contained in MRS medium is preferential adapt for utilization of some strains of bacteria and propagate quickly, which is helpful in establishing a new micro ecosystem after cultivation at 37°C under facultative anaerobic conditions (40). This resulted in significant differences in the bacterial composition between cultured and uncultured flushing liquids of the heart in metagenomic sequencing.

Mice reared at 37°C experienced heat stress and oxidation in organs due to the environment. *Ligilactobacillus salivarius* and *Lactobacillus reuteri* are the core bacteria for body inherited from ancestor (19), which is substantial in maintaining nutritional absorption and immunity. Dietary supplementation *Ligilactobacillus salivarius* can improve the bacterial abundance of the GIT and its bodily immune and anti-oxidative role in animals (22, 41, 42). The mice were gavage administration of *Ligilactobacillus salivarius* to determine the bacterial composition. The results suggested that *Ligilactobacillus salivarius* supplementation enriched the bacterial composition in the ileum, liver, and heart of mice. At the phylum level, Bacillota and Pseudomonadota were abundant instead of Bacteroidota in these organs. The ratio of Bacillota to Pseudomonadota increases, which is helpful for absorbing nutrition, improving immunity, and anti-oxidation in organs (43). Heat and administration of *Ligilactobacillus salivarius* were the two primary factors that played key roles in bacterial differences at both the phylum and genus levels. Mice with *Ligilactobacillus salivarius* supplementation enhanced the bacterial diversities, more genera of *Lactobacillus*, *Paenibacillus*, norank_f_*Mitochondria*, and unclassified_k_norank_ d_*Bacteria* detected in heart, which functioned in immunity and anti-oxidation (37, 44, 45). At the species level, *Lactobacillus reuteri* and *Burkholderia stabilis* and *Ralstoniapickettii* improved in hearts and livers, which play key roles in anti-oxidation defense.

The 16S rDNA sequencing results indicated that mice supplemented with *Ligilactobacillus salivarius* had an increased abundance of *Lactobacillus* sp, wherein the number of *Lactobacillus reuteri* was most improved in the heart, liver, and ileum compared with that in control mice. In the gut, nutrition originates from the digestion of chyme, which fulfills the requirements for the propagation of abundant strains of bacteria (46). Gastrointestinal bacterial composition is influenced by several factors. The richness of *Lactobacillus reuteri* and other functional bacteria improved in mice administration of *Ligilactobacillus salivarius* in heat stress. However, in the heart and liver, nutrition primarily originates from sterile tissue fluids and blood. The origin of the increased supply of bacteria, such as *Lactobacillus reuteri*, *Burkholderiastabilis*, and *Ralstoniapickettii* even some non-intrinsic bacteria, in the heart or liver, is unknown. *Lactobacillus reuteri* is the core bacterium residing in the gastrointestinal tract (47) also other certain strains of bacteria namely *Lactobacillus plantarum* (48), *Lactobacillus murinus* (49), and *Bifidobacterium* sp (50), and functions as a substantial microbiota in the microecosystem. Our results suggest that a greater number of *Lactobacillus reuteri* can be found in both the heart and liver. These unusual bacteria were observed to be the core species residing in the GIT (35, 36), suggesting that they migrate from the gut to the lungs to defend against unfavorable conditions. During heat exposure, the composition of the microbiota changes, which can be restored and optimized through oral supplementation with probiotics in humans and animals to alleviate the damage caused by oxidation (51, 52). The gut is a key source of bacteria in the body. From the results of this study, we hypothesize that organs encountering the stress can send distress signals to initiate the translocation of intestinal core bacteria to other organs, which is a “cry for help” phenomenon. This “cry for help” phenomenon also occurred in plant enrolled the rhizomicrobiome to defend against diseases and insect pests in the surface (53). *Lactobacillus reuteri* is a core bacterial strain that is trans-located to enrich the composition of heart and liver, which is helpful to defend against heat stress in organs. Some other core bacteria strains of *Lactobacillus* and *Bifidobacterium* also be enrolled to aid the liver and heart. The selective bacteria must following the enrolled signals called by requirements of aided organs for certain needed. Considering the trans-located pathway of pathogens was through circulated system (54), the destroyed blood barrier or lymph circulation would the potential road to help the trans-location (55). It is speculated that the pathway for transportation of intestinal core bacteria was initiated by the gut-heart axis (5) and gut-liver axis (6) and the bacteria were transported through the lymphatic circulation (56).

Translocation of the core bacterium *Lactobacillus reuteri* from the gut enhanced the bacterial composition to improve its anti-oxidative role in the heart and liver. However, the signals and pathways of translocation remain unclear and require further study. Also, when body was threatened by other stressors, such as cold, exposure of ammonia, or other harmful gas, infection of pathogens, the cry for help of core bacteria from gut to aid the crisis can still be occurred, which need to be further investigated.

## 5 Conclusion

Our findings revealed the presence of intrinsic bacteria in the heart and liver. The bacterial composition can be optimized by the translocation of core bacteria from the GIT by oral supplementation with the probiotic *Ligilactobacillus salivarius*, which induces higher anti oxidative capacities and defends the heart and liver against heat stress. Meanwhile, the choice of certain core bacteria as a probiotic supplement is crucial for clinical use. The study provides insights into the translocation of core bacteria in response to responding of the needs of the heart and liver in addition to the gut to enrich their intrinsic microbiota to defend against heat stress.

## Data Availability

16S sequencing data have been deposited into the Sequence Read Archive database (SRP) of NCBI (SRR18190482). The BioProject accession number is PRJNA811797. Culturomics sequencing data on heart has been deposited into the Sequence Read Archive database (SRP) of NCBI (SRR29254823). The BioProject accession number are PRJNA1119040. The whole genetic sequencing data of heart, liver and lung have been deposited into the Sequence Read Archive database (SRP) of NCBI (SRR29213176, 29234301, 29240348). The BioProject accession number are PRJNA1117782, 1118213, 1118345.

## Supplementary material

Supplementary Table 1 The first animal assay. Seventy-two specific pathogen free male BALB/c mice were all in control group. The aim was to verify the existence of intrinsic microbiota in the organs of normal health mice.

Supplementary Table 2 The second animal assay. *Ligilactobacillus salivarius* was cultured and diluted with saline. The live number reached 1×10^8^ CFU/mL.

Supplementary Table 3 PCR primers.
