# Supplementary material for: Ligilactobacillus salivarius regulating translocation of core bacteria to enrich mouse intrinsic microbiota of heart and liver in defense of heat stress
Source: Front Immunol. 2025 Apr 10;16:1540548. doi: 10.3389/fimmu.2025.1540548 (PMC12018310; doi:10.3389/fimmu.2025.1540548)
Supplement: Supplementary Table 1 — The first animal assay. Seventy-two specific pathogen free male BALB/c mice were all in control group. The aim was to verify the existence of intrinsic microbiota in the organs of normal health mice. [file Table1.doc]

**Supplementary table 1 The first animal assay**

| Treated | Feeding Duration | Total number | Sampled number |
| --- | --- | --- | --- |
| Normal adaptive feeding | 7 d | 72 | 10 |

Seventy-two specific pathogen free male BALB/c mice were all in control group. The aim was to verify the existence of intrinsic microbiota in the organs of normal health mice.

**Supplementary table 2 The second animal assay**

| Group | Gavaged volume | Live bacteria |
| --- | --- | --- |
| *Ligilactobacillus salivarius* supplemented | 0.4 | 4×10^7^ CFU/mL |
| Control | 0 | 0 |

*Ligilactobacillus salivarius* was cultured and diluted with saline. The live number reached 1×10^8^ CFU/mL.

**Supplementary table 3 PCR primers.**

| Gene name | Forward 5’-3’ | Reverse 5’-3’ |
| --- | --- | --- |
| *Primer of qPCR of L. reuteri* | AACTCCCTGAAATGACAGTGAAG | TGACTGAACACTAACCCGAACCT |
| *Probe for FISH of L. reuteri* | CCGAGTTGAGAGACTGATCGGCCACAATGGAACTGAGACACGGTCCATACTCCTACGGGAGGCAGCAGTAGGGAATCTTCCACAATGGGCGCAAGCCTGATGGAGCAACACCGCGTGAGTGAAGAAGGGTTTCGGCTCGTAAAGCTCTGTTGTTGGAGAAGAACGTGCGTGAGAGTAACTGTTCACGCAGTGACGGTATCCAACCAGAAAGTCACGGCTAACTACGTGCCAGCAGCCGCGGTAATACGTA | |
